# Lassa fever: another threat from West Africa

**DOI:** 10.1186/s40696-016-0018-3

**Published:** 2016-04-30

**Authors:** Tal Brosh-Nissimov

**Affiliations:** Israel Defense Forces Medical Corps, Tel Hashomer, Israel

**Keywords:** Lassa fever, Lassa virus, Arenavirus, Ribavirin, Bioterrorism

## Abstract

Lassa fever, a zoonotic viral infection, is endemic in West Africa. The disease causes annual wide spread morbidity and mortality in Africa, and can be imported by travelers. Possible importation of Lassa fever and the potential for the use of Lassa virus as an agent of bioterrorism mandate clinicians in Israel and other countries to be vigilant and familiar with the basic characteristics of this disease. The article reviews the basis of this infection and the clinical management of patients with Lassa fever. Special emphasis is given to antiviral treatment and infection control.

## Background

Lassa fever (LF), or Lassa hemorrhagic fever, is an infection caused by Lassa virus (LASV). This fairly common disease, endemic to West Africa, is associated with significant morbidity and mortality in some cases, and is contagious. Hospitalized patients with LF may pose a substantial risk to healthcare workers (HCWs) and to other patients. The recent major outbreak of Ebola virus disease (EVD) in West Africa, together with a few cases of EVD importations to countries outside Africa [1–5] and nosocomial transmission to HCWs [2, 6], raised the vigilance of western nations for EVD and similar contagious infections [7]. In the meanwhile, a few large scale outbreaks of LF were simultaneously reported from Nigeria since August 2015, with a cumulative suspected case count of 159 as of January 2016 [8]. International transportation to and from Africa increased dramatically in the last decade, further increasing the risk for infectious disease exportation from endemic areas [9]. Unlike the rare outbreaks of EVD, LF cases in Africa are common and occur annually, therefor posing a continuous theoretical threat to travelers. Thus, local physicians in Israel and in other countries outside Africa should be familiar with LF as a possible diagnosis in travelers. The article will review the updated data about LF epidemiology, pathogenesis and clinical management.

## Methods

A Pubmed search for medical literature was carried out using the terms “Lassa fever” and “Lassa virus”, and “Lassa” together with the terms “Travelers”, “Importation”, “Healthcare workers”, “Laboratory acquired infection”. Additional data was retrieved from the World Health Organization (WHO) and Centers for Disease Control and Prevention (CDC) websites. Relevant publications were reviewed and are presented herein.

### Virology

LASV is a single-stranded RNA virus, belonging to the arenaviruses (AV) family. All members of this family are composed of two segments of ambisense RNA and a nucleoprotein (NP), surrounded by a lipid envelope and a glycoprotein (GP). Electronic microscopy shows characteristic grainy particles inside the virus, traced to be host ribosomes, which give it its name (Arena = sandy).

The AV are classified according to their geographic distribution. The old world AV include the world-wide leukocytic choriomeningitis virus (LCMV), and the African LASV, Lujo virus, and a few other viruses, not known to be pathogenic to humans. The new world AV is distributed in specific areas in the American continents, and includes the pathogenic Junin, Guanarito, Machupo, and Sabia viruses and other non-pathogenic species [10].

### Epidemiology

The AV are sustained in nature in chronically infected rodents. The natural host of LASV is *Mastomyces natalensis*, the most common rat in rural West Africa, commonly found in households. The virus is shed in the rat’s urine, feces, and respiratory secretions and is found in blood. Humans get infected by direct contact with the rat’s excretions, by inhaling dust contaminated with it, or by eating the rat [11, 12]. Person to person transmission occurs by direct contact, and there is very little epidemiological support for significant airborne transmission [13, 14]. Contagiousness begins with symptom onset and increases with disease severity, consistent with the appearance of pharyngeal shedding, vomiting, diarrhea and bleeding, and increasing levels of viral load in body fluids [15–17]. The virus is shed in the urine for 3–6 weeks, and up to 3 months in semen, with risk for sexual transmission, prompting condom use in survivors [11, 12, 14, 18]. Cumulative experience in the western world, with more than 25 imported cases and more than 1500 potential contacts, showed a very limited risk of transmission, with only one suspected asymptomatic infection [16].

The disease is endemic in Nigeria, Liberia and Sierra Leone, with seroprevalence rates of 7 % to more than 20 % [10, 13]. Proven cases or seropositivity were also reported in Cote d’Ivoire, Guinea, Central African Republic, Mali, Senegal and Congo [18]. The annual incidence is estimated as 100,000–300,000 cases, out of which 5000 are fatal [12]. These regions are also endemic to other hemorrhagic fever viruses, including Ebola, and indeed an outbreak of LF occurred in Liberia during 2014, as the activity of EVD was high, complicating the differential diagnosis of suspected cases [19]. As the incubation period can be fairly long, and the clinical presentation is non-specific, LF is a potential imported infection in travelers from endemic countries, even though the incidence is low, with about 27 cases reported so far [16, 17, 20–24]. The last two cases were reported from the US in 2014 and 2015 [23, 24]. Of 24 cases with reported data, almost all have worked in an endemic area for a prolonged time, with a third as HCW’s, and five as aid-workers or peace-keepers. Seven of these died [22]. Some of the patients were medically evacuated from Africa, but others travelled by themselves, sometimes after disease presentation [17, 21].

### Pathogenesis

LASV has a broad cell tropism, mostly using a cytoskeleton associated peptide, α-dystroglycan, as its receptor. After infection the virus proliferates mainly in macrophages, dendritic and endothelial cells. The infection does not lead to lytic damage, and pathogenesis is related to immune suppression, uninhibited viral proliferation and host responses. LASV inhibits host immune response in various ways. It bypasses the usual route of endosomal trafficking, crucial for innate immunity recognition. The viral NP directly suppresses interferon production, and infected immune cells do not secrete other pro-inflammatory cytokines such as TNF-α, IL-6, and IL-1β. Therefore, LF does not manifest with a “cytokine storm” as other hemorrhagic viral fevers [13, 25].

The most important pathological change is an increase in capillary permeability, with development of edema and hypovolemic shock. Other changes include hepatitis with hepatic necrosis, and necrosis of the spleen and adrenals [13, 25].

The immune response to LASV is not completely understood. Cellular immunity is most important, with strong T cell responses in survivors [10]. Antibody responses are probably less important, and although specific antibodies are produced early in the disease, neutralizing antibodies appear only after weeks or months with low titers and avidity [11].

### Clinical manifestation

The incubation period is usually 7–10 days, with a reported range of 3–21 days [11, 13, 15, 16, 18]. Twenty percent of cases have a severe disease, requiring hospitalization, while 80 % have a mild or asymptomatic infection. The general case fatality rate is estimated as 1–2 %. The mortality rate of hospitalized cases in Africa is 15–20 % [13], with reports of up to 50 % in some outbreaks [26].

The disease begins gradually with a nonspecific flu-like illness, including fever and malaise. After 1–3 days, the patients report headache, throat pain, myalgia, abdominal pain, a retrosternal chest pain, cough, diarrhea, and vomiting. Physical examination might reveal hypotension, exhudative pharyngitis, lymphadenopathy, conjunctivitis, and a maculopapular rash. Severe LF usually manifests in the second week with hypovolemic shock, facial edema, pulmonary edema, pleural effusion, ascites, renal failure, and neurological signs such as confusion and seizures. Hemorrhage is found in 17 % of cases only, is limited to mild mucosal bleeding, and does not significantly contribute to shock. Survivors show improvement within 8–10 days, while fatal cases progress to coma, shock and death during the third week [10, 11, 13, 18]. Risk factors for death include age of <18 years or elderly, neurological involvement, pharyngitis, vomiting, aspartate transaminase (AST) level above 110 IU, hemorrhagic signs and a plasma viral load of more than 10^3.6^ TCID_50_ (50 % tissue culture infective dose) [10, 11, 13, 18].

Laboratory findings include increase in transaminases, proteinuria, leukopenia, anemia and mild thrombocytopenia, with a significant disorder of platelet function. Coagulation studies are usually normal [27].

The clinical presentation of LF is very similar to other common and endemic African diseases, such as malaria, typhoid fever, rickettsial infections and influenza, complicating the differential diagnosis of returning travelers. Travelers with mild infections might be diagnosed if clinicians would be aware of disease activity in the origin country, and therefore updates of the activity of LF and other contagious infections are important for clinicians in Israel and other countries. Symptoms that were found to be predictive of LF are fever, retrosternal chest pain, pharyngitis and proteinuria [18], with the first three predicting LF diagnosis with a positive predictive value of 81 % [28].

Various complications were reported, including pericarditis, arrhythmia, pancytopenia and renal failure. Central nervous system involvement is typical, with development of meningitis, encephalitis, encephalopathy, and cerebellar ataxia. Virus was isolated from CSF in some cases [29]. Sensorineural hearing loss (SNHL) is very typical and common, reported in 13.5 % of acute cases, and predicting worse outcome. Its presence during illness or recrudescence is suggestive of LF. After recovery, a third of patients will have SNHL, irreversible in two-thirds of them [30, 31]. Severe abdominal pain with peritoneal signs was reported, with many cases operated for suspected surgical and gynecological emergencies. Some of these cases led to surgical staff infection [32]. LF tends to be more severe during pregnancy, mainly in its late stages, with fatality rates of up to 50 % and fetal loss in 75–100 % of cases [13, 26]. Uterine evacuation can lead to dramatic improvement [18, 33].

### Diagnosis

The most useful way for diagnosis is polymerase chain reaction (PCR) from blood. Sensitivity was reported as 79 % on the first day of hospitalization, increasing to 100 % on the third day [11, 13, 18]. Genetic strain variation might rarely lead to false negative results [34], and laboratory contamination to false positive ones [35]. Various serological tests are being used, including direct NP antigen testing and specific IgG and IgM antibodies against NP and GP. A mixed NP and IgM ELISA has a sensitivity and specificity of 88 and 90 %, respectively [18]. IgM can persist for months and years, and IgG for decades [36]. Cross reactions with LCMV exist [17]. Clinical samples from LF patients are a significant hazard to laboratory personnel, with percutaneous inoculations and contact with mucosal surfaces as the main risk factors for infection. This mandates high level of safety in collection and processing of samples, including use of personal protective equipment by laboratory personnel. Isolation of LASV in cell cultures and in animals is the gold standard, but necessitates extreme biosafety conditions (Biosafety level 4) [37].

### Treatment

Similar to other severe hemorrhagic fevers, supportive treatment is the cornerstone of clinical management of LF. The main goal is volume resuscitation, accounting for third spacing, diarrhea and vomiting, while avoiding volume overload due to the risk of pulmonary edema. Other goals are electrolyte balance and respiratory support.

Convalescent plasma, although beneficial in some animal experiments, has failed clinical studies, probably due to lack of neutralizing antibodies [38, 39]. Ribavirin, a broad spectrum guanosine analogue antiviral, possesses good activity against LASV. Intravenous treatment in standard doses leads to plasma concentrations that are significantly higher than the minimal inhibitory concentration (MIC), while oral treatment, limited by side effects and a 50 % bioavailability, leads to low to borderline concentrations, doubtfully inhibiting LASV in vivo [16]. Animal studies with parenteral ribavirin treatment proved it to be protective, with survival benefit in non-human primates, even when treatment was begun 5 days after infection [16]. A controlled clinical trial performed in the 1980s by the CDC in Sierra Leone, assessed the benefit of intravenous and oral ribavirin [39]. The results of this single human trial are presented on Table 1. Ribavirin treatment was significantly associated with survival. Benefit was even more significant in higher risk patients with high level viremia and increased liver function tests, and when within 6 days from symptom onset. Both oral and intravenous ribavirin were beneficial, with the latter showing a stronger effect in higher risk cases. The recommended intravenous dosing is based on this pivotal study, with a 2.4 g loading dose, followed by 1 g every 6 h for 10 days, for average weight adults.

**Table 1:** Mortality rate in LF patients treated with ribavirin [39]

Risk group	Treatment onset (days from symptom onset)	Mortality rate		
		No treatment (%)	Oral ribavirin (%)	Intravenous ribavirin (%)
AST >150 IU	All cases	55	14*	19*
	Within 6 days	61	20*	5*
	After >7 days	52	11*	26*
Viremia >10^3.6^ TCID_50_	All cases	76	30*	32*
	Within 6 days	75	20*	9*
	After >7 days	78	40*	47*
Viremia <10^3.6^ TCID_50_	All cases	28	7*	9*

*AST* aspartate transaminase, *TCID*_*50*_ 50 % Tissue culture inhibitory dose

* Significantly lower than mortality with no treatment

Ribavirin’s main adverse effect is dose dependent hemolysis, appearing in ~20 % of patients, usually resulting in modest decrease in hematocrit [39]. Oral treatment is associated with many more adverse effects, including nausea, vomiting, diarrhea, metal taste, dry mouth, myalgia, fatigue, headache, jaundice, rash, tachycardia, thrombocytosis, increased lipase levels, mood changes and insomnia. However, no mortality was reported after ribavirin treatment [16]. Ribavirin is teratogenic and embryotoxic in rodents, and is contraindicated during pregnancy and lactation, although due to the grave prognosis of LF in pregnant women, the risk should be weighed against its benefit.

Various experimental treatments were evaluated for LV, including antivirals, molecules targeting host cells, and immunomodulators. None is currently clinically proven or approved for treatment [40].

### Prevention

The main preventive step in endemic areas is rodent control in and around dwellings, avoiding contact with rats and consumption of them [13]. For foreign workers and diplomats living in endemic areas during outbreaks, the main preventive strategies are to avoid contact with rodents, with ill persons and with local health services, if proper infection control practices are not well maintained. No vaccine is currently available against LASV. Effective vaccine candidates should produce long lasting strong cellular immune responses. Various vaccines were tested in animals [38], including live virus vaccine with non-pathogenic AV such as Mopeia virus, and recombinant viral vectors such as vaccinia virus, vesicular stomatitis virus and yellow fever virus, carrying LASV antigens.

When dealing with imported cases in the western world, the main focus for prevention is hospital infection control, with extensive history of LF nosocomial outbreaks in Africa. The main risk is from contaminated needles or direct contact of patient blood or secretions with mucous membranes or injured skin. These outbreaks involved institutions with very low level of infection control, lack of protective equipment (gloves, masks), needle re-use, and surgery under poor hygienic conditions [10, 41]. Indeed, in African hospitals, where proper barrier nursing was practiced, HCW’s did not have a higher seroprevalence for LASV than the neighboring rural community [42]. In addition, only one suspected secondary infection of a HCW was reported from cases treated outside Africa, with a physician exhibiting LASV-IgG without symptoms after inserting an intravenous catheter without gloves into a severe LF case [17]. Airborne transmission of LASV is not proven. Only one report from Nigeria in 1970, suggested airborne transmission within a hospital, with secondary cases that did not have direct or close contact with the original case [43]. The virus is stable in aerosols under conditions of low temperature and humidity, and successfully infects animals via the airborne route [11, 44]. Transmission of LASV between distanced animal cages was reported, too [45]. Due to these factors, LASV is categorized as a high risk (Biosafety level 4) agent for laboratory work [37], but the recommendations for hospital infection control are paradoxical. Formal international guidelines from the WHO include only contact and droplet-based precautions, with use of gloves, long sleeved coats, surgical mask and a face/eye shield [14]. Others recommend negative-pressure isolation rooms [11], or specialized high-level isolation units, using exceptional infection control practices, in order to prevent risks to HCW’s [46, 47]. In fatal cases, special care of patient’s remains should be practiced in order to prevent exposure to body fluids or tissues. This might include incineration or closed coffins, and avoiding direct contact with the body [15].

Post exposure prophylaxis (PEP) with ribavirin after contact with a LF patient can be offered. Animal studies have shown good protection up to 5 days post-exposure. No clinical study was done to evaluate PEP efficacy. Most of the data is anecdotal, and actually the oral regimens that were used are expected to produce lower ribavirin plasma levels than those proven effective in animal studies by an order of magnitude [16]. Since oral ribavirin treatment is associated with a high rate of adverse effects, PEP is recommended only to high risk contacts: contaminated needle accidents, direct contact of body fluids with mucous membranes or injured skin, participation in emergency medical treatment without proper protective equipment or staying with the patient in an enclosed space for many hours. Consideration can be given to the patient’s level of contagiousness, reflected by the severity of illness and presence of vomiting, diarrhea and bleeding. A dosing protocol was suggested by Bausch et al. [16] with a 2.4 g loading dose, followed by 1 g every 6 h for 6 days, and 0.5 g every 6 h for 4 more days.

### Conclusions

Physicians in Israel and other countries outside Africa have to be alert to the risk of imported endemic disease, such as LF. The ever-growing global travel is increasing the risk for such an episode. In addition, LASV can serve as an agent of bioterrorism, leading to a local outbreak in non-endemic areas [48]. It is categorized as a category A threat priority agent by the US National Institute of Allergy and Infectious Diseases (NIAID) [49]. Military physicians might be especially involved if military personnel are used for management of bioterrorism incidents or if soldiers are deployed to endemic regions.

Although usually non-fatal and not highly contagious, some patients develop a severe infection and lead to nosocomial outbreaks. Clinicians should suspect LF in travelers from an endemic area with a febrile illness, after ruling out common travel-related infections. Unlike most other viral hemorrhagic fevers, LF can be treated with ribavirin, if diagnosed early enough. Simple good infection control principles will prevent most of the risk for nosocomial transmission. The use of airborne precautions, probably unnecessary for most LF patients, might be considered in severe cases, and while conducting aerosol-generating procedures.
